# Apoptotic effects of hsian‐tsao (*Mesona procumbens* Hemsley) on hepatic stellate cells mediated by reactive oxygen species and ERK, JNK, and caspase‐3 pathways

**DOI:** 10.1002/fsn3.1046

**Published:** 2019-04-22

**Authors:** Yung‐Hsiang Yeh, Chun‐Ya Liang, Mao‐Liang Chen, Fu‐Ming Tsai, Yi‐Ying Lin, Ming‐Cheng Lee, Jiunn‐Sheng Wu, Chan‐Yen Kuo

**Affiliations:** ^1^ Division of Gastroenterology Chang Bing Show Chwan Memorial Hospital Changhua Taiwan; ^2^ Department of Medical Research and Development Chang Bing Show Chwan Memorial Hospital Changhua Taiwan; ^3^ Department of Research, Taipei Tzu Chi Hospital Buddhist Tzu Chi Medical Foundation New Taipei City Taiwan; ^4^ Division of Infectious Diseases Taipei Tzu Chi Hospital Buddhist Tzu Chi Medical Foundation New Taipei City Taiwan

**Keywords:** hepatic stellate cell, hsian‐tsao, reactive oxygen species, apoptosis

## Abstract

The activation of hepatic stellate cells (HSCs) is an important step in the progress of liver fibrosis. Fibrosis can be impeded by HSC reversion to a quiescent state or HSC clearance through apoptosis. To investigate the apoptotic effects of hsian‐tsao (*Mesona procumbens* Hemsl) on human HSCs, the expression levels of cleaved caspase‐3, p38, and c‐Jun N‐terminal kinase (JNK) were assessed using Western blotting, and the caspase‐3 activity was measured using caspase‐3/CPP32 colorimetric assay kit. Hsian‐tsao extract (HTE) increased the activity of caspase‐3 and the level of activated caspase‐3, indicating the activation of apoptosis. The intracellular reactive oxygen species (ROS) level increased in a dose‐dependent manner. This increase was prevented by an antioxidant, suggesting that HTE induces ROS accumulation. In addition, we found that HTE induced the phosphorylation of the mitogen‐activated protein kinases JNK and p38. These collective data indicate that HTE induces apoptosis via ROS production through the p38, JNK, and caspase‐3‐dependent pathways. HTE may decrease HSC activation in liver fibrosis and may have a therapeutic potential.

## 1 INTRODUCTION

Liver fibrosis is associated with severe morbidity and significant mortality (Bonis, Friedman, & Kaplan, 2001), and it involves the activation of hepatic stellate cells (HSCs) (Friedman, 2008a, 2008b). In liver fibrosis, the activated HSCs undergo proliferation, which can result in the inhibition of apoptosis, the accumulation of extracellular matrix (ECM), and the production of proinflammatory proteins (Friedman, 2010; Murphy et al., 2002). Therefore, HSCs are believed to be the key target for fibrosis treatment (Fallowfield, 2011; Friedman, 2008b). It has been reported that decreasing the survival rate of activated HSCs can be achieved via the inhibition of cell proliferation or by triggering apoptosis; in addition, this can be achieved by the suppression of excessive ECM deposition (Fan et al., 2013). Thus, the idea to decrease the activated HSC survival rate by using natural products could be effective in the treatment of liver fibrosis.

*Mesona procumbens* is a natural drink and is the main component of grass jelly in Taiwan (Huang et al., 2012). *M. procumbens* has therapeutic potential in the treatment of inflammation‐associated disorders (Huang et al., 2012). The inhibition of monosodium urate‐induced xanthine oxidase activity in human acute monocytic leukemia THP‐1 cells by a 50% ethanol extract of *M. procumbens* has been demonstrated (Jhang et al., 2016); this highlights the potential to improve hyperuricemia by the downregulation of xanthine oxidase activity in vivo. Aqueous extracts of hsian‐tsao have been reported to protect the myocardium in streptozotocin‐induced diabetic rats (Yang et al., 2008). Analysis of the serum levels of hepatic enzymes in experimental animal models revealed that the aqueous extracts of hsian‐tsao protect against tertiary butyl hydroperoxide‐induced acute hepatic damage and reduce oxidative stress (Yen, Yeh, & Chen, 2004). Many reports have indicated that reactive oxygen species (ROS) play a key role in the regulation of the activation of mitogen‐activated protein kinases (MAPKs), such as p38 and c‐Jun N‐terminal kinase (JNK) (Chuang & Chen, 2004; Jia et al., 2007; Junttila, Li, & Westermarck, 2008; McCubrey, Lahair, & Franklin, 2006; Son, Kim, Chung, & Pae, 2013).

However, the pharmacological effects and the mechanism of action of HTE on the inhibition of liver fibrosis are still unknown.

Herein, we report that a hsian‐tsao extract from *M. procumbens* Hemsl has an apoptotic effect on activated HSCs via ROS and the p38 MAPK, JNK, and caspase‐3‐dependent pathways.

## 2 MATERIALS AND METHODS

### 2.1 Materials

Dried hsian‐tsao leaves were purchased from BioWisdom. Water extraction of hsian‐tsao was performed as described by Yang et al. (2008). Finally, the final extract was collected and used for the experiments.

### 2.2 Reagents

A WST‐1 kit was purchased from Roche Applied Sciences. A caspase‐3/CPP32 colorimetric assay kit was purchased from BioVision. The antiphospho‐JNK and JNK antibodies were purchased from Cell Signaling Technology. The antiphospho‐p38, p38 MAPK, and glyceraldehyde 3‐phosphate dehydrogenase (GAPDH) antibodies were purchased from Santa Cruz Biotechnology. Sigma‐Aldrich was the manufacturer of all other chemicals.

### 2.3 Cell culture

Human primary hepatic stellate cells (hHSCs) were obtained from ScienCell Research Laboratories and were cultured according to the manufacturer's instructions. Briefly, the cells were seeded into poly‐l‐lysine‐coated T‐25 flasks in Stellate Cell Medium (ScienCell Research Laboratories) containing 2% fetal calf serum (FCS) and stellate cell growth supplement (ScienCell Research Laboratories).

### 2.4 Detection of cell viability and caspase‐3 activity

We used a WST‐1 cell proliferation assay kit and caspase‐3/CPP32 colorimetric assay kit to detect the cell viability and caspase‐3 activity in this study, respectively. The protocols were supplied by the manufacturer and were modified according to our previous study (Kuo et al., 2014).

### 2.5 Intracellular ROS analysis

Fluorescence‐activated cell sorting (FACS, BD Biosciences) was used to detect the relative ROS levels after the cells were stained with the reagent 2′,7′‐dichlorofluorescein diacetate (DCF‐DA; Sigma‐Aldrich).

### 2.6 Western blotting

The cell was harvested and lyzed according to a protocol from our previous study (Kuo et al., 2014). The primary antibodies were used and are mentioned in the above section (2.2. Reagents).

### 2.7 Statistical analyses

All data were examined by one‐way or two‐way analysis of variance (ANOVA). Additionally, the Bonferroni post hoc test was used in this study. A *p*‐value <0.05 was reported as statistically significant. *p < 0.05, **p < 0.01.

## 3 RESULTS

### 3.1 HTE treatment decreased the cell viability via apoptosis

**Figure 1:**
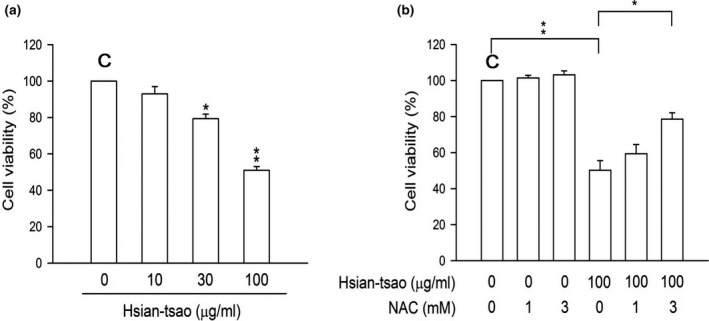
Hsian‐tsao extract (HTE) decreases the cell viability (a) Cells were treated with the indicated concentrations of hsian‐tsao for 24 hr; the control cells were not treated. (b). N‐acetyl‐cysteine (NAC) (3 mM) reversed hsian‐tsao‐induced cell death. The experiments were independently repeated three times (*n* = 3). C: means control group. *p < 0.05, **p < 0.01

**Figure 2:**
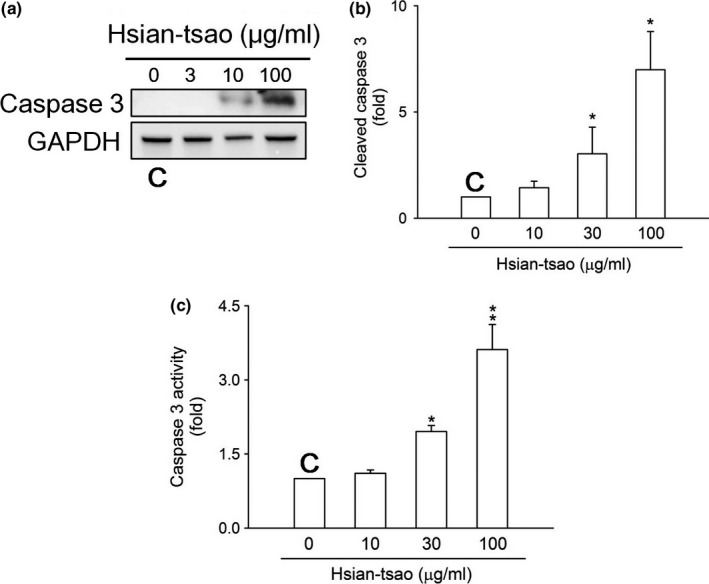
Hsian‐tsao extract (HTE) induces cell death via caspase‐3 activation (a) Cells were treated with the indicated concentrations of HTE or were left untreated (control) for 24 hr. The level of activated caspase‐3 (cleaved caspase‐3) was analyzed using Western blotting. Glyceraldehyde 3‐phosphate dehydrogenase (GAPDH) was used as a loading control. (b) The expression of specific proteins was quantified using ImageJ. (c) Cells were untreated (control) or treated with the indicated concentrations of hsian‐tsao for 24 hr. C: means control group. The experiments were independently repeated three times (*n* = 3)

The cell viability was inhibited in a dose‐dependent manner with HTE treatment (10–100 μg/ml) (Figure 1a). To assess the apoptotic effects on HSCs, we evaluated the level of activated caspase‐3 (cleaved caspase‐3) and the activity of caspase‐3 by Western blotting and enzyme‐linked immunosorbent assays, respectively. The data demonstrate that both levels were significantly increased in cells treated with HTE (30 and 100 μg/ml) for the indicated time periods (Figure 2a–c). We hypothesize that HTE can induce apoptosis via a caspase‐3‐dependent pathway.

### 3.2 Intracellular ROS production was induced under HTE treatment

**Figure 3:**
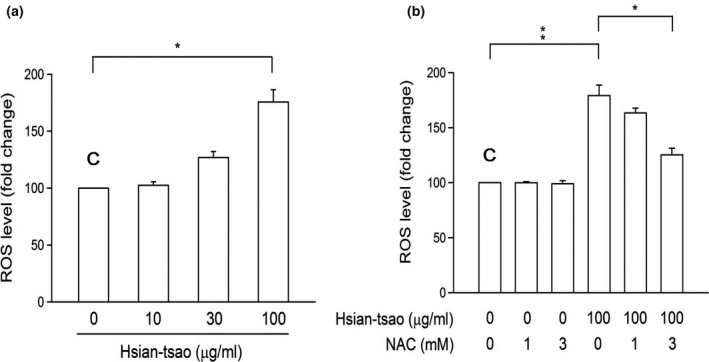
Hsian‐tsao extract (HTE) induces reactive oxygen species (ROS) production (a) Cells were treated with hsian‐tsao at the indicated concentrations for 24 hr. After DCF‐DA staining, fluorescence‐activated cell sorting (FACS) detected and quantitated the fluorescence signal. (b) N‐acetyl‐cysteine (NAC) (3 mM) reversed the induced ROS overproduction. The experiments were independently repeated three times (*n* = 3). C: means control group

DCF‐DA staining showed that HTE (100 μg/ml) significantly induced ROS production (Figure 3a). Furthermore, treatment with the ROS scavenger N‐acetyl‐cysteine (NAC, 3 mM) attenuated HTE‐induced ROS production (Figure 3b) and cell death (Figure 1b).

### 3.3 Effect of HTE on the phosphorylation of JNK and p38 MAPK

**Figure 4:**
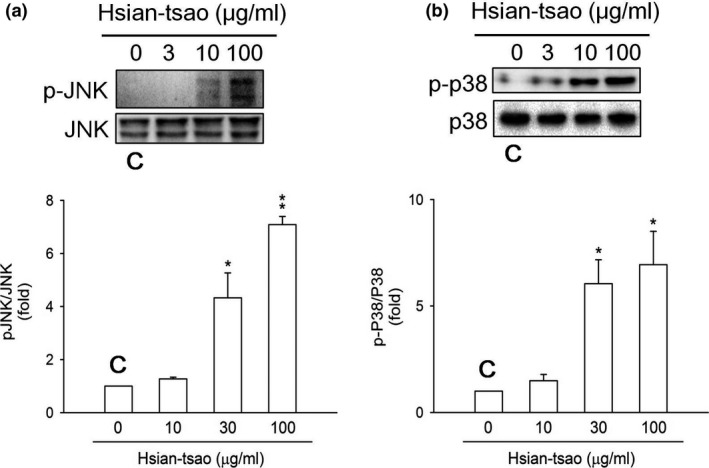
Hsian‐tsao extract (HTE) induces the phosphorylation of JNK and p38. The induced phosphorylation of (a) JNK and (b) p38 in a dose‐dependent manner. The phosphorylation of JNK and p38 was quantitated using antibodies against the phosphorylated and total protein. Densitometric analysis was carried out by normalizing the total protein levels (lower panels of a and b). The experiments were independently repeated three times (*n* = 3). C: means control group

**Figure 5:**
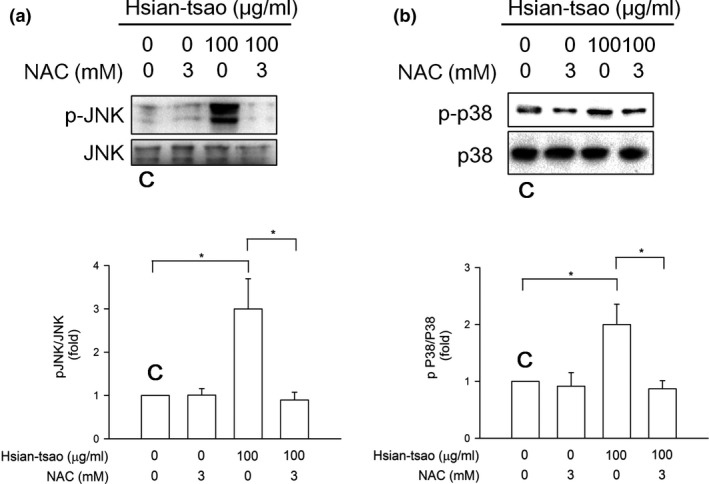
N‐acetyl‐cysteine (NAC) reverses the hsian‐tsao extract (HTE)‐induced phosphorylation of JNK and ERK. The NAC reversal of the HTE‐induced phosphorylation of (a) JNK and (b) p38. Cells were pretreated with NAC (3 mM) for 1 hr before HTE treatment. The expression levels of phospho‐JNK, JNK, phospho‐p38, and p38 were detected with Western blotting. Densitometric analysis of all samples was carried out by normalizing the total protein levels. The experiments were independently repeated three times (*n* = 3). C: means control group

**Figure 6:**
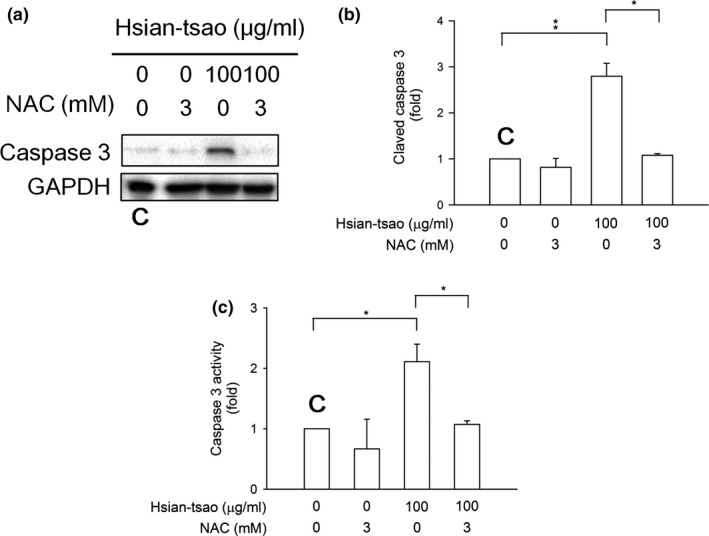
N‐acetyl‐cysteine (NAC) reverses hsian‐tsao‐induced caspase‐3 activation. (a) Cells were pretreated with NAC (3 mM) for 1 hr before the addition of hsian‐tsao extract (HTE). The Western blot analysis showed the level of activated caspase‐3 (cleaved caspase‐3). Glyceraldehyde 3‐phosphate dehydrogenase (GAPDH) was used as a loading control. (b) The expression of specific proteins was quantified using ImageJ. (c) Caspase‐3 activity was detected. The experiments were independently repeated three times (*n* = 3). C: means control group

The JNK and p38 pathways have been implicated in cell apoptosis (Troeger et al., 2012). To examine whether JNK and p38 phosphorylation is associated with HTE ‐induced apoptosis, the expression and phosphorylation of both proteins were measured by Western blotting. HTE rapidly induced JNK and p38 activation in time‐ and concentration‐dependent manners (Figure 4a,b). This phosphorylation was reversed by treatment with 3 mM NAC (Figure 5a,b). The increases in the cleaved caspase‐3 expression level and in caspase‐3 activity were also reversed after NAC treatment (Figure 6). These results suggest that HTE causes cell apoptosis due to ROS overproduction.

## 4 DISCUSSION

The activation of HSCs is a key role in liver fibrosis (Franco & Cidlowski, 2012). Therefore, increasing the apoptotic levels of activated HSCs and decreasing the growth rate of activated HSCs may be good strategies for solving the problem of liver fibrosis (Friedman, 2010; Kuo et al., 2014; Mederacke et al., 2013; Puche, Saiman, & Friedman, 2013; Ray, 2014; von Schwarzenberg & Vollmar, 2013). Drugs that inhibit hepatic fibrogenesis might be obtained from marine natural products. To the best of our knowledge, this is the first study to demonstrate an apoptotic effect of the natural product HTE on HSCs via ROS accumulation.

We investigated the pharmacological effects of HTE on HSCs using multiple approaches and found that it induces apoptosis through ROS production via the p38, JNK, and caspase‐3‐dependent pathways. Many studies have demonstrated the role of HSC activation in liver fibrosis and have highlighted the significance of HSC‐induced apoptosis in the pathogenesis of liver fibrosis (Friedman, 2010; Issa et al., 2001; Jia et al., 2015; Kuo et al., 2014; Mederacke et al., 2013; Puche et al., 2013; von Schwarzenberg & Vollmar, 2013; Xie, Fujii, Zhao, Shinohara, & Matsukura, 2016). Therefore, we hypothesize that hsian‐tsao may potently inhibit HSC viability through ROS production via the JNK and p38 MAPK pathways.

N‐acetyl‐cysteine can prevent JNK phosphorylation in human gastric carcinoma MKN45 cells (Guo et al., 2016) and can reverse the overexpression of p38‐associated pathways in vascular endothelial cells (Bhattacharya, Halder, Mukhopadhyay, & Giri, 2009) and human melanoma cells (Bell et al., 2010). It has been reported that Pin was isolated from the gorgonian coral *Pinnigorgia* sp., which triggered the activated HSCs to undergo apoptosis via ROS‐ERK/JNK‐caspase‐3 signaling and probably caused the clearance of HSCs (Kuo et al., 2018). Another study suggested that the traditional Chinese medicine *Fuzheng Huayu* attenuates hepatic fibrosis by inhibiting tumor necrosis factor‐alpha‐induced hepatocyte apoptosis and by activating HSCs in mice treated with carbon tetrachloride (Tao et al., 2014). Curcumol is a guaiane‐type sesquiterpenoid hemiketal extracted from the roots of the herb *Rhizoma Curcumae*. It has been shown to target receptor‐interacting serine/threonine‐protein kinase‐1/‐3 and to induce necroptosis in HSCs via JNK1/2‐ROS signaling cascades (Jia et al., 2018). These data clearly show that natural products can protect against liver fibrosis through the deactivation of HSCs or the induction of cell death.

Accumulating evidence has suggested that ROS play a critical role in the progression of liver fibrosis formation (Ceni, Mello, & Galli, 2014; Parola & Robino, 2001; Poli & Parola, 1997; Siegmund et al., 2007). ROS accumulation caused cell death via apoptosis in activated HSCs in both humans and rats (Brunati, Pagano, Bindoli, & Rigobello, 2010). To further characterize the HTE‐induced cell apoptotic pathway, we studied the effect of HTE on ROS‐induced cell death and determined the origin of ROS production. The 1‐glutathione precursor NAC reversed the apoptosis induced by HTE and the cell death resulting from ROS accumulation (Figures 1b, 3b, and 6). Thus, we hypothesize that HTE induces apoptosis by ROS overproduction and through l‐glutathione depletion. These findings are consistent with the present and prior findings (Dunning et al., 2009, 2013; Gao et al., 2012; Kuo et al., 2014; Runchel, Matsuzawa, & Ichijo, 2011; Zarubin & Han, 2005).

ERK, p38 kinase, JNK, and MAPK are members of the MAPK family and are important for the response to oxidative stress (Chowdhury et al., 2013; Huang, Wu, Tashiro, Onodera, & Ikejima, 2008; Zarubin & Han, 2005). It has been reported that the survival of activated HSCs is mediated by the MAPK signaling pathway (Jia et al., 2018; Szuster‐Ciesielska, Mizerska‐Dudka, Daniluk, & Kandefer‐Szerszen, 2013). However, Yu et al. (2012) found that the continuous generation of hydrogen peroxide may result in the inhibition of the growth of human gingival fibroblasts and that this effect is independent of MAPK activation. Therefore, the mechanism underlying the MAPK‐mediated apoptosis of HSCs induced by oxidative stress is still unclear.

Currently, phenolic compounds (kaempferol, apigenin, caffeic acid, protocatechuic acid, syringic acid, vanillic acid, and *p*‐hydrobenzoic acid) were extracted from hsian‐tsao (Yeh, Huang, & Yen, 2009). Kaempferol inhibits pancreatic cancer cell growth and migration through the blockade of an EGFR‐related pathway (Lee & Kim, 2016). Apigenin has health‐promoting effects or therapeutic functions for chronic diseases, including diabetes, amnesia, Alzheimer's disease, depression, insomnia, and cancer (Salehi et al., 2019). Caffeic acid has anti‐inflammatory, anticancer, and antiviral activities (Touaibia, Jean‐Francois, & Doiron, 2011). On the other hand, protocatechuic acid displays notable atheroprotective effects via the regulation of the transition of M1‐ and M2‐type macrophages (Liu et al., 2019). The protective effect of syringic acid on myocardial infarction (MI) caused by isoproterenol (ISO) has been reported (Shahzad et al., 2019). Vanillic acid has potential as an agent for the treatment of sickle cell anemia and chronic liver injuries (Itoh et al., 2009; Safo & Kato, 2014). *p*‐Hydrobenzoic acid has antimicrobial, antialgal, antimutagenic, antiestrogenic, hypoglycemic, anti‐inflammatory, antiplatelet aggregating, nematicidal, antiviral, and antioxidant functions; in addition, it is widely used in cosmetic products (Azam, Dharanya, Mehta, & Sachdeva, 2013). Therefore, these phenolic compounds extracted from hsian‐tsao have antioxidant and anti‐inflammatory effects.

Herein, JNK and p38 were activated significantly after HTE treatment (Figure 4). Consistent with the previous results, the apoptotic effect of shikonin was determined in leukemia cells via ROS and the JNK pathway (Mao, Yu, Li, & Li, 2008). Panaxydol induces cellular apoptosis via the JNK/ROS pathway (Kim et al., 2011). Thus, the present study shows that the signaling pathway underlying HTE‐induced apoptosis in HSCs involves ROS production and JNK and p38 activation.

## 5 CONCLUSIONS

We have shown a significant induction of apoptosis in HSCs by hsian‐tsao treatment. These apoptotic effects are mediated through multiple mechanisms: the increased accumulation of ROS and the activation of JNK and p38. The structures of the bioactive compounds in HTE remain to be elucidated. In conclusion, our study makes a significant contribution toward the identification of pathways which can be pharmaceutical targets for the development of novel therapeutic approaches in the treatment of liver fibrosis.

## CONFLICT OF INTEREST

None declared.

## ETHICAL STATEMENT

This study does not involve any human or animal experiments.
